# Dissecting the Interactions of Diabetes Mellitus and Hearing Loss with Cognitive Decline and Dementia

**DOI:** 10.3390/brainsci15070669

**Published:** 2025-06-21

**Authors:** Sofia Waissbluth, Paul H. Delano

**Affiliations:** 1 Department of Otolaryngology, Pontificia Universidad Católica de Chile, Santiago 8330033, Chile; 2 Department of Neuroscience, Facultad de Medicina, Universidad de Chile, Santiago 8380453, Chile; pdelano@hcuch.cl; 3 Centro Avanzado de Ingeniería Eléctrica y Electrónica, AC3E, Universidad Técnica Federico Santa María, Valparaíso 2390123, Chile; 4 Servicio de Otorrinolaringología, Hospital Clínico Universidad de Chile, Santiago 8380456, Chile

**Keywords:** cognition, brain, hearing loss, diabetes mellitus, cochlea, dementia

## Abstract

The aging population is increasingly affected by both diabetes mellitus and hearing loss, two conditions that often coexist and can significantly impact quality of life. As the prevalence of diabetes rises with age, so does the incidence of hearing impairment, with both conditions contributing to cognitive decline and functional limitations. The interplay between diabetes, hearing loss, and cognition highlights the need for comprehensive healthcare strategies that address the unique challenges faced by older adults. This review explores the mechanisms underlying the interplay between these three conditions, including mitochondrial dysfunction, neuroinflammation, oxidative stress, and microangiopathy.

## 1. Introduction

**Figure 1:**
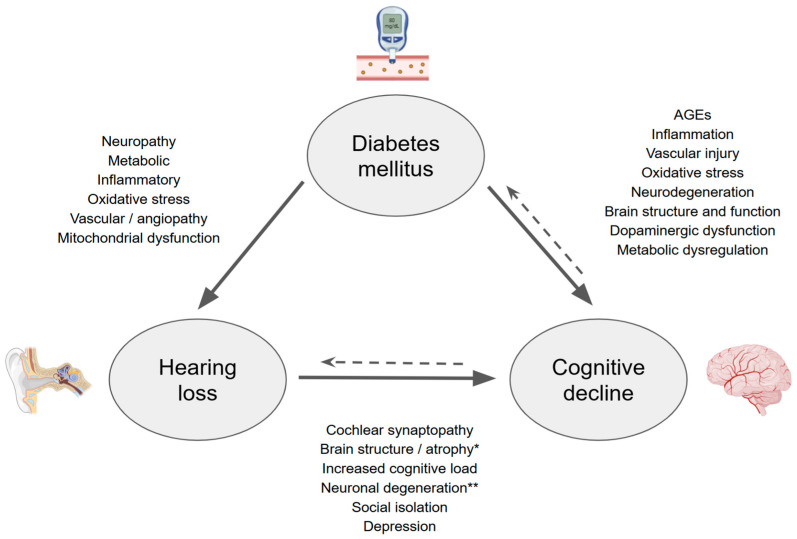
Potential mechanisms interlinking between diabetes mellitus, hearing loss, and cognitive decline. Many factors contribute to hearing loss and cognitive impairment. Mitochondrial dysfunction and oxidative stress contribute significantly to the pathophysiology of both hearing loss and dementia. Type 2 diabetes mellitus can cause hearing loss by microangiopathy, neuropathy, oxidative stress, and mitochondrial dysfunction, which can affect the cochlea and auditory pathways. Hearing loss can further impact dementia by central and peripheral mechanisms, as well as social isolation and depression. AGEs: advanced glycation end products. * Temporal lobes and other regions associated with auditory processing and cognitive [5,6]; ** neuroinflammation and tau protein phosphorylation in the hippocampus [7]. How cognitive decline could impact diabetes incidence is beyond the scope of this article.

Diabetes mellitus and hearing loss are common disorders affecting hundreds of millions of people worldwide, particularly among aging populations [1]. Both conditions are significant risk factors for cognitive decline and dementia, which currently affect nearly 57 million individuals [2]. Recent studies suggest a potential interplay between these three conditions, highlighting how diabetes may exacerbate hearing loss, and vice versa [3], while also influencing cognitive function [4]. Both diabetes and hearing impairment are known to be associated with neurodegenerative processes, but the mechanisms linking them to cognitive decline remain under investigation. Understanding the complex relationships between these factors could provide valuable insights into their combined impact on overall health, offering opportunities for targeted interventions aimed at improving quality of life and reducing healthcare burdens (Figure 1). Diabetes mellitus-associated cognitive decline may be mediated by hearing impairment-related pathways and may share common pathophysiological mechanisms as hearing loss-associated cognitive decline. This review explores the emerging evidence regarding the interconnections between hearing loss, diabetes, and cognitive function, emphasizing the need for a multidisciplinary approach in addressing these interconnected challenges.

## 2. Hearing Loss and Its Relationship with Cognition

Over 1.5 billion people worldwide are affected by some form of hearing loss. Among them, around 430 million individuals have moderate or severe hearing loss in their better ear [8]. The prevalence of hearing loss differs across World Health Organization (WHO) regions, with the majority of those impacted residing in low- and middle-income countries [8]. The prevalence of hearing loss varies depending on the type, which includes sensorineural, conductive, mixed, and central auditory dysfunction. Sensorineural hearing loss, which results from damage to the inner ear or auditory nerve, is the most prevalent form, particularly among older adults, where age-related hearing loss (ARHL) is widespread. ARHL affects over 60% of individuals by the age of 70 and approximately 80% of those over 85 years of age [9,10]. Conductive hearing loss, caused by issues in the outer and/or middle ear, is less common but still notable, especially in pediatric populations in low- and middle-income countries, where childhood hearing loss affects approximately 1% of children [11]. Mixed hearing loss, characterized by the coexistence of sensorineural and conductive components, is less frequently observed than the other types [11]. Central auditory dysfunction, which involves auditory and non-auditory brain circuits, is less commonly discussed in prevalence studies but is recognized as a distinct category of hearing impairment [12,13]. The literature does not provide specific prevalence data for central auditory dysfunction, but it is understood to be less common than peripheral types of hearing loss [14]. Overall, hearing loss is a multifaceted condition with varying prevalence across different types and populations, influenced by factors such as age, socioeconomic status, and geographic location [8].

Hearing loss can arise from damage to the cochlea, auditory nerve, ribbon synapses, spiral ganglion neurons, and synapses between the hair cells and nerve terminals, as well as damage to or dysfunction of the auditory brainstem or cerebral cortex, which can affect the processing of auditory information [15]. Each type of hearing loss has distinct pathophysiological mechanisms and clinical implications, necessitating tailored diagnostic and management approaches [16,17,18,19,20,21]. The loss of inner hair cells (IHCs) has a major impact on the auditory nerve by disrupting the synaptic connections between IHCs and auditory nerve fibers. These cells are essential for transmitting sound information to the brain, as they form synapses with 90–95% of type I auditory nerve fibers. When these synapses are lost, cochlear synaptopathy can occur, often due to factors like noise exposure, aging, or genetics. This results in a decrease in neural output from the cochlea, but hearing thresholds in quiet environments may remain unaffected [22,23,24]. As a result of IHC and synapse loss, there is a reduction in the compound action potential produced by type I neurons, which reflects the overall neural activity in the cochlea. The remaining auditory nerve fibers can still maintain frequency tuning and low thresholds, suggesting that some compensatory mechanisms are in place within the auditory system [24]. Despite this, the compensation is typically inadequate in more challenging listening situations, such as understanding speech in noisy environments, where higher behavioral thresholds and increased masking effects are observed [24]. Additionally, the loss of IHCs and their synapses may lead to changes in how the central auditory system processes sound. Research indicates that the central auditory system may increase its sensitivity in response to diminished cochlear input, a phenomenon referred to as central auditory gain [24]. Central auditory gain refers to a compensatory process that occurs when there is a reduction in afferent input from the auditory nerve, with heightened cortical activity despite a decrease in peripheral input. This can lead to central auditory system hyperexcitability. In older adults, the reduced afferent input from the auditory nerve is linked to lower levels of cortical GABA, a neurotransmitter involved in inhibitory signaling. The decline in GABA contributes to an increase in central gain, which, in turn, can impair speech recognition in noisy environments [25]. The central nervous system compensates for reduced afferent input by amplifying the strength of central auditory responses. However, amplifying the response may not lead to improvement in auditory processing difficulties. Neural synchrony deficits could explain this problem. A recent study investigating synchrony and aging found that synchrony declines with age in both auditory nerve and midbrain responses. Additionally, it was observed that age-related central gain takes place without a corresponding improvement in inter-trial synchrony [26]. While central compensation can help restore normal hearing in quiet conditions, it may also cause difficulties in more complex auditory tasks and potentially contribute to conditions like hyperacusis [24,27].

The loss of IHCs results in a weakened auditory signal, which demands greater cognitive effort to process, particularly in difficult listening conditions like noisy environments. This heightened cognitive load can place a strain on executive functions and working memory, as individuals need to dedicate more mental resources to comprehend speech. As a consequence, fewer cognitive resources are left available for other tasks, potentially impacting overall cognitive performance [13,28,29]. The literature suggests that hearing loss, including IHC loss, is associated with changes in brain structure and function of auditory and non-auditory regions [30,31,32,33]. There is evidence of neurochemical and functional reorganization in the auditory and cognitive networks, which may contribute to cognitive impairment. Specifically, decreased levels of auditory gamma-aminobutyric acid (GABA)-mediated inhibition [24] and altered connectivity between auditory and cognitive networks have been observed, which are linked to reduced speech perception and cognitive performance [25,34]. Additionally, hearing loss has been associated with cortical atrophy, including reductions in whole-brain cortical thickness and hippocampal volume, which are critical for cognitive processing [5,6,35]. Moreover, hearing loss can lead to compensatory changes in the brain, where cognitive cortical areas may attempt to compensate for the impaired auditory processing. This compensatory mechanism, however, may not fully mitigate the cognitive decline associated with hearing loss, as it can lead to further cognitive strain and potential decline in cognitive reserve [36].

Overall, the interplay between hearing loss and cognitive function is complex, involving both direct effects on auditory processing and indirect effects through increased cognitive load and neural reorganization. This highlights the importance of addressing hearing loss as a modifiable risk factor for cognitive decline and underscores the potential benefits of interventions such as hearing aids to mitigate these effects [10].

## 3. What About Hearing Loss and Dementia?

There is evidence for an association between hearing loss and dementia [37,38]. Dementia is a clinical condition characterized by a significant decline in one or more cognitive functions, which severely impacts an individual’s ability to perform daily activities. It is typically an acquired disorder that affects various cognitive domains, such as memory, language, attention, visuospatial abilities, and executive function, often accompanied by mood disturbances [39,40]. The diagnostic process for dementia includes a thorough assessment, incorporating a detailed medical history, cognitive and physical evaluations, and, in some cases, neuroimaging and laboratory tests to determine potential underlying causes [39]. Dementia can be categorized into distinct subtypes based on the specific pathological mechanisms involved. Alzheimer’s disease (AD) is the most prevalent form; other subtypes include vascular dementia, mixed dementia, and rarer forms such as frontotemporal dementia and dementia with Lewy bodies [41,42,43].

**Figure 2:**
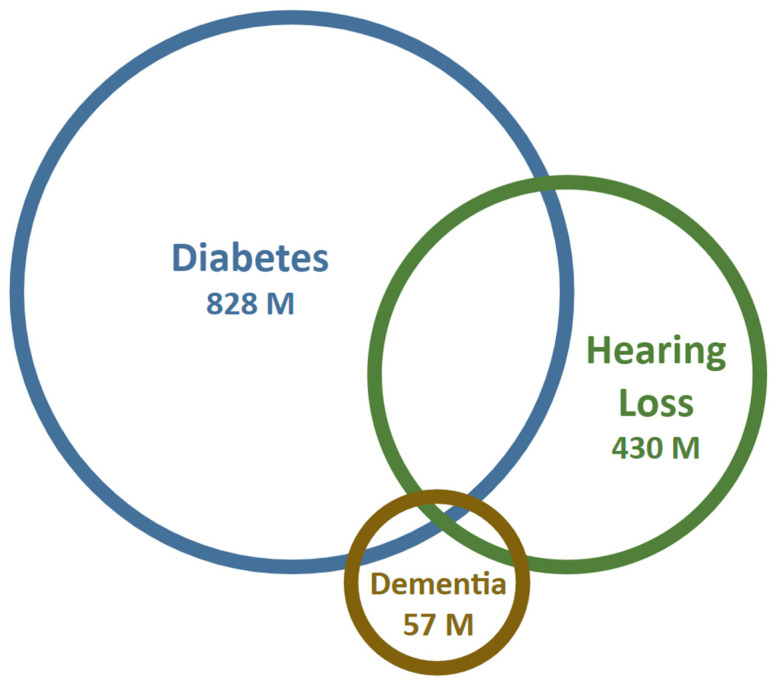
Prevalence of diabetes, hearing loss, and dementia. Type 2 diabetes mellitus (T2DM) and age-related hearing loss are highly prevalent conditions, with an overlap ranging from 20% to 60% according to various cohort studies (Table 1). In contrast, dementia is less prevalent and shows approximately a 10% overlap with diabetes. The sizes of the circles represent population size. Data taken from WHO reports on diabetes, hearing loss, and dementia. These estimates are approximations, as the diagnostic criteria for hearing loss, the degrees of cognitive decline, and the stages of dementia vary considerably. This variability complicates the ability to define a precise overlap between hearing loss, dementia, and diabetes mellitus. Therefore, we present general trends rather than exact figures.

The global prevalence of dementia is considerable and continues to rise. Recent estimates suggest that approximately 47 million people worldwide are affected by dementia, with projections indicating that this number could increase to 131 million by 2050 [39]. The WHO reported that, in 2021, 57 million people worldwide lived with dementia (Figure 2) [2]. The prevalence of dementia is influenced by factors such as geographic region, age, and gender. A systematic review reported a pooled prevalence of all-cause dementia in individuals aged 50 years and older at 697 per 10,000 persons, with AD and vascular dementia affecting 324 and 116 individuals per 10,000 persons, respectively [41]. Notably, the prevalence is higher in women compared to men and escalates with advancing age [44]. These findings emphasize the increasing global public health burden associated with dementia, including associated risk factors.

Hearing loss is increasingly recognized as a significant modifiable risk factor for dementia, with a robust body of evidence supporting this association [45]. Multiple studies have demonstrated a notable correlation between hearing impairment and an elevated risk of developing dementia. An analysis utilizing data from the South Korean National Health Information Database revealed that the severity of hearing loss is associated with an increased risk of all types of dementia, with hazard ratios indicating a heightened risk for individuals with severe hearing loss [46]. Additionally, a systematic review and meta-analysis of cohort studies found that adult-onset hearing loss is linked to an increased risk of developing cognitive impairment and dementia, including AD. However, this association was not observed for vascular dementia [47].

**Table 1:** Incidence of hearing loss in diabetic (T2DM) patients vs. controls.

Study	Sample (Control/Diabetic)	Age (Control/Diabetic)	Incidence of Hearing Loss (Control/Diabetic)
ElSherif M et al. (2024) [48]	40/42	49.7 ± 6.4/50.7 ± 6.4	0%/9.5%
Alizadeh Y et al. (2022) [49]	105/315	60.0 ± 8.8/59.8 ± 8.2	Control: 10.2% Diabetic: 11.6% (no DR) * 12.9% (mild–mod NPDR) 34.2% (severe NPDR/PDR)
Mishra A & Poorey (2019) [50]	50/50	Age-matched	18%/74%
Li J et al. (2018) [51]	43/51	54.4 ± 10.1/56.1 ± 10.1	25.6%/45.1%
Adebola S et al. (2016) [52]	90/97	58.8 ± 14.7/58.9 ± 14.9	8.9%/21.5%
Bamanie A & Al-Noury K (2011) [53]	87/109	45.7/47.9	39.1%/69.7%
Mozaffari M et al. (2010) [54]	80/80	45.1/45	20%/45%
Aladag I et al. (2009) [55]	37/63	47.5/46.6	48.6%/44%
Mitchell P et al. (2009) [56]	1648/210	69.7/70.5	38.2%/50%
Sakuta H et al. (2007) [57]	596/103	52.9 ± 1.0	45.2%/60.2%

* DR: diabetic retinopathy; NPDR: non-proliferative DR (NPDR); PDR: proliferative.

Lin and Albert (2014) [58] proposed aging and microvascular disease as possible common etiologies linking hearing loss and cognitive decline. In this context, mitochondrial dysfunction, oxidative stress, and neurodegeneration can play significant roles in the association of hearing loss and dementia. Mitochondrial dysfunction plays a crucial role in both sensorineural hearing loss and neurodegenerative diseases, including AD. The cochlea, due to its high metabolic activity, is particularly dependent on mitochondrial function for energy production. Mitochondrial dysfunction disrupts energy metabolism and increases the generation of reactive oxygen species (ROS), which contributes to cellular damage in both cochlear structures and brain regions involved in cognitive processes [59,60]. This dysfunction is implicated in the degeneration of cochlear cells, such as sensory hair cells and spiral ganglion neurons, which are vital for auditory processing [60]. On the other hand, oxidative stress also appears to be a common pathological mechanism underlying both hearing loss and neurodegeneration. In the cochlea, oxidative stress can result from various factors, including noise exposure, aging, and ototoxicity, leading to damage of cochlear structures and the subsequent development of hearing impairment [61]. Similarly, oxidative stress is a prominent feature in neurodegenerative diseases, including AD, where it contributes to neuronal damage and cognitive decline [62,63]. The shared pathways of oxidative stress suggest a potential mechanistic link between hearing loss and dementia, where heightened oxidative stress in both the auditory and cognitive systems may exacerbate neurodegenerative processes [62], leading to the progressive loss of neuronal integrity and function. In hearing loss, neurodegeneration primarily affects the auditory pathways, with evidence also pointing to dysfunction in the hippocampus. In contrast, dementia involves neurodegeneration in brain regions essential for memory and cognitive processing [64]. Individuals with hearing impairment often rely on additional cognitive resources—such as executive and attentional networks—to compensate for reduced auditory input. This increased listening effort and cognitive demand may gradually exhaust cognitive reserve, diminishing the brain’s resilience to further neurological damage and potentially hastening cognitive decline [10,28].

Overall, mitochondrial dysfunction and oxidative stress contribute significantly to the pathophysiology of both hearing loss and dementia, offering a biological foundation for their association. The cochlea’s vulnerability to oxidative stress, particularly in the mitochondria, is a key factor in sensorineural hearing loss. Understanding these underlying mechanisms underscores the potential for therapeutic interventions, such as antioxidant-based therapies, to slow the progression of both conditions [62]. Another important aspect to consider is the amyloid pathology observed in AD. Amyloid plaque accumulation in the auditory cortex and brainstem regions, including the inferior colliculus and medial geniculate body [65], have been associated with enhanced central auditory gain [66]. Interestingly, transgenic AD mice (5xFAD mouse model), exhibit alterations in auditory processing, specifically an elevation in central auditory gain, which precedes other auditory impairments such as hearing loss and diminished auditory brainstem response wave I amplitude [66]. The prognostic value of central auditory processing was assessed prospectively in humans, and a central auditory speech-processing deficit assessed using the Synthetic Sentence Identification with Ipsilateral Competing Message test showed a positive predictive value for subsequent probable AD of 47% [67]. Auditory evoked potentials have been highlighted as potential non-invasive biomarkers for AD, reflecting neurodegenerative processes in the central nervous system that precede significant cognitive decline [66,68,69].

Other researchers have also emphasized the potential of auditory evoked potentials as non-invasive biomarkers for AD. Auditory evoked potentials, which reflect neural responses to auditory stimuli, have been shown to exhibit alterations in individuals with AD and in AD mouse models, suggesting their utility in detecting early disease-related neurophysiological changes. These findings further support the role of central auditory processing assessments in the early diagnosis of AD. Studies have shown that hearing loss can lead to changes in brain structure, such as gray matter atrophy [7]. Brain changes in the temporal regions, which are critical for auditory and speech processing, as well as the frontal lobe areas related to attentional control, have been associated with hearing loss [70]. Recently, Paciello et al. describe that noise-induced auditory damage affects the hippocampus, causing memory deficits in a model of early age-related hearing loss. Interestingly, we now know that the hippocampus in dementia undergoes significant structural and functional changes, including atrophy, loss of synapses, and disruption of memory processes, which contribute to the cognitive deficits and memory loss characteristic of dementia. Paciello et al. observed that hippocampal synaptic alterations in noise-exposed animals are associated with redox imbalance and neuroinflammation [71]. The role of sensorineural hearing loss as a predictor of dementia highlights the importance of addressing hearing loss as a potential strategy to mitigate cognitive decline. An important meta-analysis published by Liang et al., investigating the association between hearing loss and the incidence of dementia [72], was published in 2021. They assessed fourteen cohorts that included 726,900 participants and found that hearing loss may increase the risk of dementia in the adult population. On the other hand, the question whether cognitive decline could lead to hearing loss arises. A biennial longitudinal study of aging demonstrated that individuals with cognitive impairment have an increased risk of subsequently reporting poor hearing, even after adjusting for multiple confounders, suggesting that cognitive decline may underlie or exacerbate hearing impairment [73].

## 4. Diabetes Mellitus and Hearing Loss

The global prevalence of diabetes mellitus (DM) has escalated markedly over recent decades. A pooled worldwide analysis estimated that approximately 828 million adults aged 18 years and older were living with diabetes, reflecting a significant increase since [74] 1990. Type 2 diabetes mellitus (T2DM) is the most prevalent form, accounting for 90–95% of all cases [75], and is characterized by relative insulin deficiency and insulin resistance. The global prevalence of T2DM is rising, driven largely by increasing rates of obesity and sedentary lifestyles. It is increasingly recognized as a risk factor for hearing loss, sharing mechanisms like oxidative stress, mitochondrial dysfunction, and inflammation, all crucial for cognitive and psychological health [76,77].

Histopathological changes in the cochlea due to DM have been observed in drug-induced diabetic animal models, genetically controlled animal studies, and human temporal bones [78]. The main findings include thickening of the basement membrane of the stria vascularis, atrophy of the stria vascularis, and progressive narrowing of the capillaries. Alterations in the vascular basement membranes are the most common findings. There is also varying susceptibility of inner ear cells to the effects of hyperglycemia [78]. Interestingly, independently of whether a patient has been treated with insulin or an oral hypoglycemic agent [79], temporal bones of T2DM patients exhibit significantly thicker walls of the vessels of the basilar membrane and stria vascularis vs. controls, as well as greater loss of cochlear outer hair cells. A meta-analysis regarding T2DM and hearing loss was published in 2014 [80]; the authors of this meta-analysis included 18 studies and reported that the incidence of hearing loss among individuals with T2DM ranged from 44% to 69.7%, which was significantly higher compared to controls. The pooled odds ratio was 1.91, indicating a nearly twofold increased risk. We expanded the search to include more recent published articles (Table 1). In all of the case–control studies, we observe that hearing loss is more prevalent in diabetic patients vs. the controls. These studies are mostly age-matched. A large prospective study published in 2017 followed 253,301 adults with normal hearing tests during a median of 4 years. They found that the rate of hearing loss in patients that had pre-diabetes and DM was greater than patients with normal glucose levels. The multivariable-adjusted hazard ratios were 1.04 and 1.36, respectively [81].

Interesting findings were reported by Alizadeh et al. with regard to concomitant retinopathy, as a known microvascular complication of diabetes. They observed that as the retinopathy was more severe, hearing loss was more common. Only one study reported similar rates between both groups, perhaps because the control group was the smallest (Aladag et al., 2009 [55]). Mitchell et al. also reported that patients suffering from diabetes for 10 or more years had worse hearing thresholds compared to those with diabetes for less than 10 years (Mitchell 2009 [56]).

Diabetic patients also exhibited abnormal wave V latencies with the auditory brainstem response test, which suggests a retrocochlear component, seeing as wave V has been attributed to the lateral lemniscus and inferior colliculus [82]. Hearing loss was more pronounced in older patients, particularly those with diabetes [80].

In diabetes, elevated blood sugar levels can trigger oxidative stress, which worsens mitochondrial dysfunction and promotes cell death in cochlear cells. This oxidative stress is driven by ROS, which can alter the stria vascularis and pericytes, ultimately resulting in hearing impairment [83]. These pericytes at the cochlear stria-blood labyrinth barrier are crucial for angiogenesis and regulating blood flow [84]. It has been recently reported that diabetes can alter the permeability of the organ of Corti, causing leakage of the stria vascularis and elevating malondialdehyde expression levels in the cochlea and Bax and caspase-3 in the stria vascularis. Pericytes exposed to a high-glucose environment exhibited an increased apoptosis rate, which increased over time, as well as a time-dependent increase in the mitochondrial ROS content in these cells [83]. A genetic mouse model that exhibits a diabetic phenotype was used in a study to evaluate cochlear synaptopathy. The authors demonstrated that the diabetic mice not only had decreased hearing (by ABR testing) but also exhibited cochlear mitochondrial dysfunction and disrupted mitochondrial structure [85]. Human studies also have shown ABR abnormalities in T2DM, with latencies and interpeak latencies being prolonged in diabetic patients [86,87]. Diabetes affects inner ear function through microangiopathy, neuropathy, oxidative stress, and mitochondrial failure. An interesting review article regarding DM and hearing loss has been written by Samocha-Bonet et al. [88].

Another association between diabetes and hearing loss is Maternally Inherited Diabetes and Deafness (MIDD), which is considered a mitochondrial disorder characterized primarily by hearing impairment and diabetes [89]. The most common reported mutation is the m.3243A>G mutation, where there is an A to G substitution at position 3243 (m.3243A>G) of the mitochondrial DNA [90]. MIDD is frequently misdiagnosed as either Type 1 or Type 2 diabetes [91]. They develop bilateral and progressive sensorineural hearing loss that begins in early adulthood [92]. Because the cochlea is highly metabolically active, a reduction in ATP resulting from mitochondrial dysfunction can lead to cell death within cochlear structures, especially the stria vascularis.

## 5. Diabetes Mellitus and Cognitive Decline

There is a recognized link between T2DM and AD, with mitochondrial dysfunction serving as a common pathological mechanism. The overlap in mitochondrial dysfunction between T2DM and AD suggests a potential mechanistic link that could contribute to the development of neurodegenerative disorders in diabetic patients. Hyperglycemia can induce oxidative stress as well as an inflammatory response, damaging cells within the central nervous system. It can also impair the integrity of the blood–brain barrier. These processes promote neurodegeneration and dementia [93]. Hyperglycemia is also linked to the formation of advanced glycation end products (AGEs). They cause dopaminergic dysfunction, and when these end products bind to their receptors, they can induce inflammation and oxidative stress and exacerbate the progression of neurodegeneration and cognitive impairment. These end products play a significant role in the development of neurodegenerative complications. An excellent review regarding potential mechanisms linking T2DM and cognitive decline and dementia progression was published by Ehtewish et al. [94]. A meta-analysis evaluating longitudinal population-based studies that assessed the association between DM and the risk of AD concluded that the risk of AD is higher among people with DM than in the general population. Data was extracted from 17 studies totaling 1,746,777 individuals [95]. An updated meta-analysis including 122 prospective studies showed that diabetics have a 43% higher risk of dementia (any type), a 43% higher risk of AD, and a 91% higher risk of vascular dementia as compared to individuals without diabetes [96]. Furthermore, a meta-analysis involving over one million individuals found that diabetics have a relative risk of 1.73 for developing dementia, 1.56 for AD, and 2.27 for vascular dementia as compared to individuals without DM [97].

An interesting finding was reported by Lin et al.; they completed a systematic review and meta-analysis regarding whether glycemic control could improve cognitive impairment in patients with DM but also for patients with hyperglycemia or insulin resistance. The three groups exhibited a significantly attenuated degree of decline in cognitive function assessment with adequate glycemic control. Hence, adequate treatment measures for DM would decrease the risk of AD [98].

Diabetes not only increases the risk of cognitive decline and AD but also alters the brain itself. Global brain atrophy has been observed in diabetic patients, and brain volume loss can be up to three times the atrophy rate of normal aging individuals. Hippocampal volumes are also decreased; however, it appears to be proportional to general brain atrophy [99].

With this vast information, we now know that DM is a risk factor for developing dementia, but is diabetes more frequent amongst populations with dementia or other cognitive impairment? The literature shows that it is. Janson et al. documented that T2DM (35% vs. 18%) and impaired fasting glucose (46% vs. 24%) were more prevalent in AD vs. controls, underscoring a strong association between the two conditions [100]. Liu et al. also found a higher prevalence of T2DM in patients with dementia (25.5%) vs. the general elderly population (15.6%) [101]. There appears to be an additional risk for women with regard to vascular dementia, but not for nonvascular dementia [102]. Collectively, these findings highlight diabetes as a modifiable risk factor for AD and other dementias.

An important point to make here is that diabetes and hearing loss are only two of the reported common risk factors for dementia. There is emerging evidence that addressing modifiable risk factors for dementia can lead to a lower likelihood of developing dementia. Besides hearing loss and diabetes, these risk factors include, education, smoking, obesity, depressive symptoms, physical inactivity, excessive alcohol intake, traumatic brain injury, exposure to air pollution, social isolation, untreated visual impairment, and elevated low-density lipoprotein cholesterol levels [45]. We understand that the interconnections are probably very vast between all these factors and that, depending on the population, one or more of these factors will predominate in determining who develops dementia. Our study aim was to explore the interconnections between hearing loss, diabetes, and cognitive function.

## 6. Conclusions

Hearing loss, T2DM, and cognitive impairment are three prevalent clinical conditions that, as demonstrated by this data, are interconnected. Further research is needed to identify the primary targets for potentially preventing the glucotoxicity associated with diabetes and dementia. As the general population lives longer, it is important to focus not only on increasing lifespan but also on ensuring a high quality of life. Gaps in the literature include prospective studies regarding whether glucose-lowering medications and hearing loss management can mitigate this risk of dementia over time and the establishment of biomarkers for patients at greater risk. Further research with regard to the role of mitochondrial dysfunction and ROS production in the pathophysiology for all three conditions should be explored. Overall, this review highlights excellent articles previously published that provide insights into DM, dementia, and hearing loss; however, we are lacking interdisciplinary research efforts to better understand the complex interplay between these conditions and elucidate the underlying neuropathological mechanisms.

## Data Availability

No new data was created or analyzed in this study.

## References

[1] GBD Results. https://vizhub.healthdata.org/gbd-results/.

[2] Dementia. https://www.who.int/news-room/fact-sheets/detail/dementia.

[3] Mittal J., Mittal R., Lemos J.R.N., Hirani K., Keith G., Lacey M., Assayed A. (2024). Diabetes mellitus, hearing loss, and therapeutic interventions: A systematic review of insights from preclinical animal models. PLoS ONE.

[4] Lee H.J., Joo Y.H., Han K.D., Park K.H. (2021). Association between hearing loss and cognitive disorder: A nationwide population-based study. Yonsei Med. J..

[5] Sugiura S., Ando F., Shimokata H., Uchida Y., Otsuka R., Nakamura A., Tange C., Iwata K., Nishita Y., Suzuki H. (2018). Smaller hippocampal volume and degraded peripheral hearing among Japanese community dwellers. Front. Aging Neurosci..

[6] Davatzikos C., An Y., Doshi J., Resnick S.M., Lin F.R., Deal J.A., Erus G., Armstrong N.M., Ferrucci L. (2019). Association of midlife hearing impairment with late-life temporal lobe volume loss. JAMA Otolaryngol. Head Neck Surg..

[7] Wang L., Feng J., Wang H.-F., Li Y., Ma Y.-H., Rolls E.T., Zhang W., Kang J., Cheng W., Yu J.-T. (2022). Hearing impairment is associated with cognitive decline, brain atrophy and tau pathology. eBioMedicine.

[8] WHO World Report on Hearing. https://www.who.int/teams/noncommunicable-diseases/sensory-functions-disability-and-rehabilitation/highlighting-priorities-for-ear-and-hearing-care.

[9] Frank L. (2024). Age-Related Hearing Loss. N. Engl. J. Med..

[10] Tsai Do B.S., Bush M.L., Weinreich H.M., Schwartz S.R., Anne S., Adunka O.F., Bender K., Bold K.M., Brenner M.J., Hashmi A.Z. (2024). Clinical practice guideline: Age-related hearing loss. Otolaryngol. Head Neck Surg..

[11] Ganek H.V., Madubueze A., Merritt C.E., Bhutta Z.A. (2023). Prevalence of Hearing Loss in Children Living in Low- And Middle-Income Countries Over the Last 10 years: A Systematic Review. Dev. Med. Child Neurol..

[12] Dubno J.R., Wilson R.H., Wingfield A., Humes L.E., Gordon-Salant S., Gates G.A., Cacace A.T., Lister J.J., Cruickshanks K.J. (2012). Central presbycusis: A review and evaluation of the evidence. J. Am. Acad. Audiol..

[13] Delgado C., Torrente M.C., Moreno-Gómez F.N., Leiva A., Marcenaro B., Belkhiria C., Martin S.S., Vergara R., Delano P.H. (2022). Speech Perception and Dichotic Listening Are Associated with Hearing Thresholds and Cognition, Respectively, in Unaided Presbycusis. Front. Aging Neurosci..

[14] Meimaroglou S., Eleftheriadis N., Iliadou V.M. (2025). Better education required for professionals in healthcare regarding auditory processing disorder. Eur. Arch. Otorhinolaryngol..

[15] Eckert M.A., Steel K.P., Schmiedt R.A., Schulte B.A., Lewis M.A., Harris K.C., Lang H., Dubno J.R., Vaden K.I. (2021). Translational and interdisciplinary insights into presbyacusis: A multidimensional disease. Hear Res..

[16] Elliott K.L., Fritzsch B., Yamoah E.N., Zine A. (2022). Age-related hearing loss: Sensory and neural etiology and their interdependence. Front. Aging Neurosci..

[17] Moser T., Starr A. (2016). Auditory Neuropathy--Neural Synaptic Mechanisms. Nat. Rev. Neurol..

[18] Rance G., Starr A. (2015). Pathophysiological mechanisms and functional hearing consequences of auditory neuropathy. Brain.

[19] Wu P.Z., O’Malley J.T., de Gruttola V., Liberman M.C. (2021). Primary neural degeneration in noise-exposed human cochleas: Correlations with outer hair cell loss and word-discrimination scores. J. Neurosci..

[20] Liberman M.C., Epstein M.J., Cleveland S.S., Wang H., Maison S.F. (2016). Toward a differential diagnosis of hidden hearing loss in humans. PLoS ONE.

[21] Shearer A.E., Hildebrand M.S., Schaefer A.M. (2023). Genetic Hearing Loss Overview.

[22] Moverman D.J., Liberman L.D., Kraemer S., Corfas G., Liberman M.C. (2023). Ultrastructure of noise-induced cochlear synaptopathy. Sci. Rep..

[23] Suthakar K., Liberman M.C. (2021). Auditory-nerve responses in mice with noise-induced cochlear synaptopathy. J. Neurophysiol..

[24] Ding D., Salvi R., Chen G.-D., Auerbach B.D., Lobarinas E., Radziwon K., Sun W., Wang J. (2017). Inner hair cell loss disrupts hearing and cochlear function leading to sensory deprivation and enhanced central auditory gain. Front. Neurosci..

[25] Harris K.C., Dias J.W., McClaskey C.M., Rumschlag J., Prisciandaro J., Dubno J.R. (2022). Afferent loss, GABA, and central gain in older adults: Associations with speech recognition in noise. J. Neurosci..

[26] Harris K.C., Dias J.W., Lang H., Panganiban C., McClaskey C.M., Noble K.V., Kerouac L.B., Rumschlag J.A. (2022). Age-related central gain with degraded neural synchrony in the auditory brainstem of mice and humans. Neurobiol. Aging.

[27] Rüttiger L., Singer W., Klose U., Wolpert S., Hofmeier B., Refat F., Wertz J., Hinrichs P., Saemisch J., Knipper M. (2021). Functional biomarkers that distinguish between tinnitus with and without hyperacusis. Clin. Transl. Med..

[28] Peelle J.E., Wingfield A. (2016). The neural consequences of age-related hearing loss. Trends Neurosci..

[29] Slade K., Plack C.J., Nuttall H.E. (2020). The effects of age-related hearing loss on the brain and cognitive function. Trends Neurosci..

[30] Doshi J., Ferrucci L., Metter E., Kraut M., Resnick S., An Y., Lin F., Davatzikos C., Goh J. (2014). Association of hearing impairment with brain volume changes in older adults. Neuroimage.

[31] Vernooij M.W., Ikram M.A., Bos D., Metselaar M., Goedegebure A., Roshchupkin G.V., Rigters S.C., de Jong R.J.B. (2017). Hearing impairment is associated with smaller brain volume in aging. Front. Aging Neurosci..

[32] Belkhiria C., Vergara R.C., Martín S. (2019). Cingulate Cortex Atrophy Associated Hearing Loss Presbycusis Cochlear Amplifier Dysfunction. Front. Aging Neurosci..

[33] Martinez M., Delano P.H., Andrade M., Leiva A., Marcenaro B., Belkhiria C., Vergara R.C., Martin S.S., Delgado C. (2020). Insula and amygdala atrophy are associated with functional impairment in subjects with presbycusis. Front. Aging Neurosci..

[34] Edden R.A., Li F., Wu L., Zong W., Ma W., Li N., Li X., Hui S.C., Ren F., Dai Z. (2023). Neurochemical and functional reorganization of the cognitive-ear link underlies cognitive impairment in presbycusis. Neuroimage.

[35] Vergara R., Delgado C., Delano P.H., García X., Vidal V., Cerda M., Navarro C.F., Leiva A., Martínez M., Belkhiria C. (2024). Cochlear dysfunction as an early biomarker of cognitive decline in normal hearing and mild hearing loss. Alzheimers Dement..

[36] Zhao F., Gao M., Feng T., Shen J., Zheng Y., Liang J., Yang H. (2023). Cognitive reserve disorder in age-related hearing loss: Cognitive cortical compensatory to auditory perceptual processing. Cereb. Cortex.

[37] Albers M.W., Gilmore G.C., Kaye J., Murphy C., Wingfield A., Bennett D.A., Boxer A.L., Buchman A.S., Cruickshanks K.J., Devanand D.P. (2015). At the interface of sensory and motor dysfunctions and Alzheimer’s disease. Alzheimers Dement..

[38] Lin F.R., Metter E.J., O’Brien R.J., Resnick S.M., Zonderman A.B., Ferrucci L. (2011). Hearing loss and incident dementia. Arch. Neurol..

[39] Arvanitakis Z., Shah R.C., Bennett D.A. (2019). Diagnosis and management of dementia: Review. JAMA.

[40] Oh E.S. (2024). Dementia. Ann. Intern. Med..

[41] Cao Q., Tan C.C., Xu W., Hu H., Cao X.-P., Dong Q., Tan L. (2020). The prevalence of dementia: A systematic review and meta-analysis. J. Alzheimers Dis..

[42] Aguilar-Navarro S.G., de la Peña J.E., Gutierez-Gutierez L., Suerna-Hernandez A., Gonzelez-Figueroa E., Juarez-Cedillo T., Garcia-Cruz J.C. (2022). Prevalence of dementia and main subtypes in Mexico: The study on aging and dementia in Mexico (SADEM). J. Alzheimers Dis..

[43] Lobo A., Launer L.J., Fratiglioni L., Andersen K., Di Carlo A., Breteler M.M., Copeland J.R., Dartigues J.F., Jagger C., Martinez-Lage J. (2000). Prevalence of dementia and major subtypes in Europe: A collaborative study of population-based cohorts. Neurologic Diseases in the Elderly Research Group. Neurology.

[44] Ren Z., Wang X.-D., Tang Y., Lv Y., Huang G., Niu J., Gang B., Meng X., Cai P., Zeng Y. (2024). The updated prevalence and risk factors of dementia in old adults in China: A cross-sectional study. J. Alzheimers Dis..

[45] Livingston G., Huntley J., Liu K.Y., Costafreda S.G., Selbæk G., Alladi S., Ames D., Banerjee S., Burns A., Brayne C. (2024). Dementia prevention, intervention, and care: 2024 report of the Lancet standing Commission. Lancet.

[46] Chang Y.-S., Lee M.K., Rah Y.C., Park S., Kim B., Choi J., Han K. (2020). Association between the severity of hearing loss and the risk of dementia within the 2010–2017 national insurance service survey in South Korea. Sci. Rep..

[47] Livingston G., Mandavia R., Omar R., Pavlou M., Lin F., Schilder A., Proctor D., Yu R.-C., Gonzalez S.C., Lewis G. (2024). Adult-onset hearing loss and incident cognitive impairment and dementia—A systematic review and meta-analysis of cohort studies. Ageing Res. Rev..

[48] ElSherif M., El Sayed Mahfouz A.F., Mohamed Gaber Amin N., Saad Kozou H., Department of Otorhinolaryngology, Audiovestibular Medicine Unit, Alexandria University Faculty of Medicine, Alexandria, Egypt (2024). Relation between glycated hemoglobin level and hearing loss in type 2 diabetic patients. J. Int. Adv. Otol..

[49] Alizadeh Y., Jalali M.M., Sehati A. (2022). Association of different severity of diabetic retinopathy and hearing loss in type 2 diabetes mellitus. Am. J. Otolaryngol..

[50] Mishra A., Poorey V.K. (2019). Clinical and audiometric assessment of hearing loss in diabetes mellitus. Indian J. Otolaryngol. Head Neck Surg..

[51] Bi J., Liu B., Zhang Y., Li Y., Li J., Fu X., Zhang L. (2018). Alteration of auditory function in type 2 diabetic and pre-diabetic patients. Acta Otolaryngol..

[52] Adebola S.O., Olamoyegun M.A., Sogebi O.A., Iwuala S.O., Babarinde J.A., Oyelakin A.O. (2016). Otologic and audiologic characteristics of type 2 diabetics in a tertiary health institution in Nigeria. Braz. J. Otorhinolaryngol..

[53] Bamanie A.H., Al-Noury K.I. (2011). Prevalence of hearing loss among Saudi type 2 diabetic patients. Saudi Med. J..

[54] Mozaffari M., Tajik A., Ariaei N., Ali-Ehyaii F., Behnam H. (2010). Diabetes mellitus and sensorineural hearing loss among non-elderly people. East. Mediterr. Health J..

[55] Aladag İ., Eyibilen A., Güven M., Atış Ö., Erkokmaz Ü. (2009). Role of oxidative stress in hearing impairment in patients with type two diabetes mellitus. J. Laryngol. Otol..

[56] Mitchell P., Gopinath B., McMahon C.M., Rochtchina E., Wang J.J., Boyages S.C., Leeder S.R. (2009). Relationship of Type 2 diabetes to the prevalence, incidence and progression of age-related hearing loss. Diabet. Med..

[57] Sakuta H., Suzuki T., Yasuda H., Ito T. (2007). Type 2 diabetes and hearing loss in personnel of the Self-Defense Forces. Diabetes Res Clin Pract..

[58] Lin F.R., Albert M. (2014). Hearing loss and dementia—Who is listening?. Aging Ment. Health.

[59] Okur M.N., Djalilian H.R. (2022). Approaches to mitigate mitochondrial dysfunction in sensorineural hearing loss. Ann. Biomed. Eng..

[60] Tan W.J.T., Song L. (2023). Role of mitochondrial dysfunction and oxidative stress in sensorineural hearing loss. Hear Res..

[61] Teraoka M., Hato N., Inufusa H., You F. (2024). Role of oxidative stress in sensorineural hearing loss. Int. J. Mol. Sci..

[62] Paciello F., Ripoli C., Fetoni A.R., Grassi C. (2023). Redox imbalance as a common pathogenic factor linking hearing loss and cognitive decline. Antioxidants.

[63] Alvarado J.C., Fuentes-Santamaría V., Juiz J.M. (2021). Frailty syndrome and oxidative stress as possible links between age-related hearing loss and Alzheimer’s disease. Front. Neurosci..

[64] Nadhimi Y., Llano D.A. (2021). Does hearing loss lead to dementia? A review of the literature. Hear Res..

[65] Weible A.P., Wehr M. (2022). Amyloid pathology in the central auditory pathway of 5XFAD mice appears first in auditory cortex. J. Alzheimers Dis..

[66] Na D., Zhang J., Beaulac H.J., Piekna-Przybylska D., Nicklas P.R., Kiernan A.E., White P.M. (2023). Increased central auditory gain in 5xFAD Alzheimer’s disease mice as an early biomarker candidate for Alzheimer’s disease diagnosis. Front. Neurosci..

[67] Gates G.A., Beiser A., Rees T.S., D’Agostino R.B., Wolf P.A. (2002). Central auditory dysfunction may precede the onset of clinical dementia in people with probable Alzheimer’s disease. J. Am. Geriatr. Soc..

[68] Shad K.F., Soubra W., Cordato D.J. (2022). The auditory afferent pathway as a clinical marker of Alzheimer’s disease. J. Alzheimers Dis..

[69] Luo Y., Liu G., Wen H., Tian X., Wei G., Wang X., Yang H., Sun S. (2024). Adjunct methods for Alzheimer’s disease detection: A review of auditory evoked potentials. J. Alzheimers Dis..

[70] McEvoy L.K., Bergstrom J., Hagler D.J., Wing D., Reas E.T. (2023). Elevated pure tone thresholds are associated with altered microstructure in cortical areas related to auditory processing and attentional allocation. J. Alzheimers Dis..

[71] Grassi C., Paludetti G., Paciello F., Pisani A., Cocco S., Rinaudo M., Fetoni A.R. (2023). Noise-induced auditory damage affects hippocampus causing memory deficits in a model of early age-related hearing loss. Neurobiol. Dis..

[72] Liang Z., Li A., Xu Y., Qian X., Gao X. (2021). Hearing loss and dementia: A meta-analysis of prospective cohort studies. Front. Aging Neurosci..

[73] de Oliveira C., Samelli A.G., de Paiva K.M., Xavier A.J., Valsechi F.E., Hillesheim D., D’oRsi E. (2023). Does cognitive impairment precede self-reported poor hearing? Results from the English longitudinal study of ageing. Int. J. Audiol..

[74] Smeeth L., Ng T.P., Zhao M.-H., Nagel G., Ulmer H., Jha A.K., Eriksson J.G., Feskens E.J., Santos R., Rigo F. (2024). Worldwide trends in diabetes prevalence and treatment from 1990 to 2022, a pooled analysis of 1108 population-representative studies with 141 million participants. Lancet.

[75] Bruemmer D., Matfin G., Polsky S., Selvin E., Beverly E.A., McCoy R.G., Lingvay I., Khunti K., Gaglia J.L., American Diabetes Association Professional Practice Committee (2025). 2. Diagnosis and classification of diabetes: Standards of care in diabetes—2025. Diabetes Care.

[76] Uchida Y., Sugiura S., Nishita Y., Saji N., Sone M., Ueda H. (2019). Age-related hearing loss cognitive decline: Potential mechanisms linking two. Auris Nasus Larynx.

[77] Livingston G., Schilder A.G., Lasica A.B., Yu R.-C., Sheppard J., Ridgway N., Omar R., Costafreda S.G. (2025). Association between adult-onset hearing loss and dementia biomarkers: A systematic review. Ageing Res. Rev..

[78] Akinpelu O.V., Ibrahim F., Waissbluth S., Daniel S.J. (2014). Histopathologic changes in the cochlea associated with diabetes mellitus—A review. Otol. Neurotol..

[79] Fukushima H., Cureoglu S., Schachern P.A., Paparella M.M., Harada T., Oktay M.F. (2006). Effects of type 2 diabetes mellitus on cochlear structure in humans. Arch. Otolaryngol. Head Neck Surg..

[80] Akinpelu O.V., Mujica-Mota M., Daniel S.J. (2014). Is type 2 diabetes mellitus associated with alterations in hearing? A systematic review and meta-analysis. Laryngoscope.

[81] Kim M.-B., Zhang Y., Chang Y., Ryu S., Choi Y., Kwon M.-J., Moon I.J., Deal J.A., Lin F.R., Guallar E. (2017). Diabetes mellitus and the incidence of hearing loss: A cohort study. Int. J. Epidemiol..

[82] Young A., Cornejo J., Spinner A. (2025). Auditory Brainstem Response.

[83] Shi T.F., Zhou Z., Jiang W.J., Huang T.L., Si J.Q., Li L. (2024). Hyperglycemia-induced oxidative stress exacerbates mitochondrial apoptosis damage to cochlear stria vascularis pericytes via the ROS-mediated Bcl-2/CytC/AIF pathway. Redox Rep..

[84] Canis M., Bertlich M. (2019). Cochlear capillary pericytes. Advances in Experimental Medicine and Biology.

[85] Lyu A.-R., Shin S.-A., Huh Y.H., Yu Y., Je A.R., Kim T.-H., Kim E.-H., Gajbhiye A., Kwon H.-C., Park Y.-H. (2021). Hearing impairment in a mouse model of diabetes is associated with mitochondrial dysfunction, synaptopathy, and activation of the intrinsic apoptosis pathway. Int. J. Mol. Sci..

[86] Sheikhzadeh M., Bagheri F., Bayani M.A., Kami M., Monadi M. (2024). Evaluation of the auditory brainstem response test in patients with type 2 diabetes mellitus. Caspian J. Intern. Med..

[87] Gupta S., Baweja P., Mittal S., Kumar A., Singh K., Sharma R. (2013). Brainstem auditory evoked potential abnormalities in type 2 diabetes mellitus. N. Am. J. Med. Sci..

[88] Samocha-Bonet D., Wu B., Ryugo D.K. (2021). Diabetes mellitus and hearing loss: A review. Ageing Res. Rev..

[89] Chanoine J.P., Thompson D.M., Lehman A. (2025). Diabetes associated with maternally inherited diabetes and deafness (MIDD): From pathogenic variant to phenotype. Diabetes.

[90] Cui Y., Liao L., Dong J., Xu L., Yang M., Jiang S., Xu C. (2021). The mutations and clinical variability in maternally inherited diabetes and deafness: An analysis of 161 patients. Front. Endocrinol..

[91] Murphy R., Turnbull D.M., Walker M., Hattersley A.T. (2008). Clinical features, diagnosis and management of maternally inherited diabetes and deafness (MIDD) associated with the 3243A>G mitochondrial point mutation. Diabet. Med..

[92] Yamasoba T., Oka Y., Tsukuda K., Nakamura M., Kaga K. (1996). Auditory findings in patients with maternally inherited diabetes and deafness harboring a point mutation in the mitochondrial transfer RNA^Leu (UUR)^ gene. Laryngoscope.

[93] Wątroba M., Grabowska A.D., Szukiewicz D. (2023). Effects of diabetes mellitus-related dysglycemia on the functions of blood–brain barrier and the risk of dementia. Int. J. Mol. Sci..

[94] Ehtewish H., Arredouani A., El-Agnaf O. (2022). Diagnostic, prognostic, and mechanistic biomarkers of diabetes mellitus-associated cognitive decline. Int. J. Mol. Sci..

[95] Zhang J., Chen C., Wang M., Hua S., Liao H., Cao F., Xiong Y. (2017). An updated meta-analysis of cohort studies: Diabetes and risk of Alzheimer’s disease. Diabetes Res. Clin. Pract..

[96] Tan L., Tan M.-S., Xue M., Cao X.-P., Ou Y.-N., Xu W., Yu J.-T. (2019). Diabetes mellitus and risks of cognitive impairment and dementia: A systematic review and meta-analysis of 144 prospective studies. Ageing Res. Rev..

[97] Gudala K., Bansal D., Schifano F., Bhansali A. (2013). Diabetes mellitus and risk of dementia: A meta-analysis of prospective observational studies. J. Diabetes Investig..

[98] Lin Y., Gong Z., Ma C., Wang Z., Wang K. (2023). Relationship between glycemic control and cognitive impairment: A systematic review and meta-analysis. Front. Aging Neurosci..

[99] Biessels G.J., Reijmer Y.D. (2014). Brain changes underlying cognitive dysfunction in diabetes: What can we learn from MRI?. Diabetes.

[100] Janson J., Laedtke T., Parisi J.E., O’Brien P., Petersen R.C., Butler P.C. (2004). Increased risk of type 2 diabetes in Alzheimer disease. Diabetes.

[101] Liu G., Li Y., Xu Y., Li W. (2022). Type 2 diabetes is associated with increased risk of dementia, but not mild cognitive impairment: A cross-sectional study among the elderly in Chinese communities. Front. Aging Neurosci..

[102] Chatterjee S., Peters S.A.E., Woodward M., Arango S.M., Batty G.D., Beckett N., Beiser A., Borenstein A.R., Crane P.K., Haan M.N. (2016). Type 2 diabetes as a risk factor for dementia in women compared with men: A pooled analysis of 2.3 million people comprising more than 100,000 cases of dementia. Diabetes Care.

